# Effect of *Radix Paeoniae Rubra* aqueous extract on the virulence and antibiotic susceptibility of multi-drug resistant *Escherichia coli*

**DOI:** 10.2478/jvetres-2026-0017

**Published:** 2026-03-30

**Authors:** Xibo Xue, Chunliang Jiang, Huixia Mao, Huiqin Duan, Ying-Jian Sun

**Affiliations:** College of Veterinary Medicine, Beijing University of Agriculture, Beijing 102206, P.R. China

**Keywords:** biofilm, drug resistant bacteria, outer membrane porin, traditional Chinese medicinal herb, virulence factor

## Abstract

**Introduction:**

Some traditional Chinese medicine herbs can be used for the treatment of infectious diseases; however, the mechanisms are not fully explored. This study aims to elucidate the mechanism of action of *Radix Paeoniae Rubra*, one of the most commonly used herbs, in bacterial infections by studying its effects on the resistance and pathogenicity of multi-drug resistant (MDR) bacteria.

**Material and Methods:**

In this study, MDR *Escherichia coli* O101 was treated with *Radix Paeoniae Rubra* aqueous extract (RPRE), and then the virulence factor protein secretion, biofilm formation and the drug resistance-related gene expression were determined by using broth microdilution, ELISA and qPCR.

**Results:**

Our results found that RPRE inhibited the bacterial virulence factor proteins. It suppressed the antibiotic-induced transcription increase of efflux pump genes *marA* and *soxS*. It inhibited not only the expression of the outer membrane porin genes *ompC* and *ompF* but also the formation of bacterial biofilms, and the combination of RPRE with antibiotics increased the bacteria’s sensitivity to them.

**Conclusion:**

Our study demonstrated that RPRE alone has a slight inhibitory effect on the bacteria, but at its sub-inhibitory concentrations, it can reduce resistance-related gene expression, inhibit virulence factor protein production and suppress biofilm formation. Furthermore, RPRE forms a bactericidal efficacy synergy when combined with antibiotics by decreasing MDR *E. coli* resistance and pathogenicity.

## Introduction

With the use of antibiotics against them, more and more bacteria have developed resistance to multiple preparations, and some bacteria have become resistant to all which are currently available (22). There is an urgent need to slow the development of resistance in pathogenic bacteria or improve the efficacy of antibiotics to extend the lifespan of the currently available ones (6). It is common knowledge that traditional Chinese medicine (TCM) has long been used for infectious diseases treatment. Compared to antibiotics, TCM drugs have advantages including fewer side effects and less likelihood of developing resistance in disease agents. *Scutellaria baicalensis, Coptis chinensis, Rhus chinensis, Akebia quinata* and *Lonicera japonica* are extracted in TCM and used for antibacterial and bacteriostatic effects (4, 9, 15, 23, 30). The mechanisms of the antibacterial properties of TCM drugs include altering bacterial membrane permeability, reducing protein and nucleic acid synthesis, inhibiting enzyme activity and controlling the growth and reproduction capabilities of pathogenic bacteria (10). *Radix Paeoniae Rubra*, also known as red paeony root and called Chi-Shao in Chinese, is a plant of the *Ranunculaceae* family and is an important medicinal herb used in TCM clinical practice for the treatment of infectious diseases (19). Our previous research found that the aqueous extract of *Radix Paeoniae Rubra* (RPRE) mainly contained a glycoside compound with a paeoniflorin nucleus, and that it could effectively inhibit the infectivity of Gram-positive *Staphylococcus aureus* (11). Whether RPRE has a similar effect on Gram-negative bacteria is not yet clear. This study mainly explored the mechanisms of RPRE in combination with antibiotics against Gram-negative *E. coli* with multi-drug resistance (MDR) and is intended to provide a theoretical basis for the application of RPRE in the treatment of MDR bacterial infections.

## Material and Methods

### Materials

*Escherichia coli* O101 was kindly provided by Professor Hong Dong from Beijing University of Agriculture. The RPRE was purchased from the TongRenTang Pharmacy (Beijing Qianmen Store, Beijing, China), and LB medium was obtained from Beijing ComWin Century Biotechnology (Beijing, China). Streptomycin, gentamicin sulphate, kanamycin, doxycycline, tilmicosin, tetracycline, cephalexin, florfenicol, spectinomycin, sulphadimethoxine and enrofloxacin were purchased from Beijing BoAoToda Technology (Beijing, China). The *E. coli* heat-stable enterotoxin (ST) and heat-labile enterotoxin (LT) ELISA kits used (cat. Nos. JL50422 and JL50416) were products of Jianglai Biotechnology Co., Ltd. (Shanghai, China). Use was made of a bacteria total RNA isolation kit (cat. No. B518625-0050) from Sangon Biotech (Shanghai, China).

### Preparation of *Radix Paeoniae Rubra* aqueous extract

The RPRE was prepared according to the reported method (11). Briefly, 200 g of *Radix Paeoniae Rubra* slices were soaked in 800 mL of deionised water for 30 min, brought to boiling point and then held just below for 30 min. The decoction was filtered and collected, and another 800 mL of deionised water was added to the *Radix Paeoniae Rubra* slices. This was repeated one more time. After two extractions, the filtered solutions were pooled, brought to boiling point and held just below for another 45 min to obtain a concentrated solution, which was cooled to room temperature and then centrifuged at 3,300 ×*g* for 15 min. The supernatant was collected and the stock solution of RPRE was prepared at a concentration of 200 mg/mL in deionised water. After aliquoting, it was autoclaved for later use; PBS was used as diluent solvent.

### Bacterial growth detection

The broth microdilution method recommended by the Clinical Laboratory Standards Institute (CLSI) (5) was used to culture the bacteria. Bacterial suspension was added to a 96-well plate at a final concentration of 1×10^5^ CFUs/mL. Sterile culture medium was used as the negative blank control, bacterial suspension without antibiotics served as the no-drug control group, and a drug control group without bacteria (a sterile control) was also set up. Different concentrations of RPRE, antibiotics or a mixture of RPRE and antibiotics were added to the *E. coli* culture medium and then incubated at 37°C for 12 h. The absorbance value (OD_600_) of the culture medium was measured by using a microplate reader. The experiment was performed three times.

The bacterial growth rate was calculated using the following formula: Bacterial growth rate (%) = (OD of drug treatment group – OD of no-drug control group) / (OD of sterile control group – OD of no-drug control group) × 100.

If the OD_600_ of the experimental group was at least 90% less than that of the no-drug control group, bacterial growth can be considered to be inhibited. The drug concentration at that point was recorded as the minimum inhibitory concentration (MIC). Breakpoints for antimicrobial susceptibility were used as given by the CLSI with regard to their specified resistance concentrations for the pair of antimicrobial and *E. coli*. Susceptible, intermediate and resistant were the three classifications cut at the breakpoints. Resistance was more finely graded as low, moderate or high also according to CLSI brackets, where *E. coli* O101 had low resistance if the determined MIC was greater than or equal to the specified resistance concentration but less than twice this concentration, it had moderate resistance if the determined MIC was greater than or equal to twice the specified resistance concentration but less than four times this concentration, and it had high resistance if the determined MIC was greater than or equal to four times the specified resistance concentration (5).

### Detection of *E. coli* virulence factor proteins

*Escherichia coli* at 1×10^6^ CFUs/mL was co-cultured with RPRE and/or antibiotics at 220 rpm and 37°C for 24 h. The culture was centrifuged at 4,000 × *g* for 10 min, the supernatant was removed and the pellet was washed three times with PBS. The pellet was then resuspended in bacterial lysis buffer and sonicated on ice for 10 min. After centrifugation at 4,000 × *g* for 10 min, the supernatant was collected and the protein concentration was measured using a BCA protein assay kit. Subsequently, the contents of heat-stable enterotoxin, heat-labile enterotoxin and endotoxin were detected using the relevant ELISA kits.

### Detection of bacterial biofilm formation

*Escherichia coli* again at 1×10^6^ CFUs/mL was co-cultured with various concentrations of RPRE and/or antibiotics at 37°C for 3 d. The bacterial suspension was then slowly aspirated, and the experimental wells were washed three times with PBS. Ice-cold methanol was added for fixation for 30 min, after which the fixative was discarded and the wells were allowed to air dry. Next, 0.1% crystal violet was added for staining for 20 min and was then removed, and the solution was washed three times with PBS. After air-drying, the biofilms were observed under a microscope and photographed. Subsequently, 95% ethanol was added to completely dissolve the dye, and the OD_570_ value was measured.

### Real-time quantitative PCR analysis

*Escherichia coli* at 1×10^6^ CFUs/mL was co-cultured with RPRE and/or antibiotics at 220 rpm and 37°C for 24 h. Bacterial total RNA was extracted using a kit and reverse-transcribed into cDNA. Primers for the nucleotide sequences of *marA, soxS, ompC* and *ompF* were designed based on GenBank sequences (Table 1), with 16S RNA as the internal reference gene, and were synthesised by Invitrogen (Thermo Fisher Scientific, Beijing, China). The PCR amplification thermal cycling conditions were as follows: pre-denaturation at 95°C for 30 s; amplification reaction at 95°C for 5 s and 58°C for 30 s for 45 cycles; and melting curve analysis at 95°C for 15 s, 60°C for 60 s and 95°C for 15 s. After the reaction was complete, the samples were stored at 4°C. The PCR data were taken for analysis of the relative gene expression using the 2-△△Ct method. Each determination was performed with four parallel samples.

**Table 1.** Primer sequences used in a qRT-PCR for analysing expression of *E. coli* O101 genes after treatment with *Radix Paeoniae Rubra* aqueous extract

Gene	Primer	Sequence (5ʹ–3ʹ)	Length (bp)
*marA*	*marA*-F *marA-*R	TTCATAGCATTTTGGACTGG ATCCGCAGCCGTAAGATGA	158
*soxS*	*soxS*-F *soxS-*R	TGACGCATCAGACGCTTGGC AAATCGGACGCTCGGTGGT	188
*ompC*	*ompC*-F *ompC-*R	GGTGGCTGGGGGGTAGATACTGG GCTTCTTTTGAATACATGGTCGATC	184
*ompF*	*ompF*-F *ompF-*R	ACCTGGCAGCGAACTACG AACATCACCGATACCTTCTACG	174
16S	16S-F 16S-R	AACTCTGTTATTAGGGAAGAACA GCTTTACGCCCAGTAATTCC	196

### Statistical analysis

Statistical analysis was performed using GraphPad Prism v. 9.0 software (GraphPad, San Diego, CA, USA). Data were expressed as means ± SD. The data were analysed with a one-way ANOVA and a Student’s *t*-test. The level of statistical significance was set at P < 0.05.

## Results

### Bacteriostatic effect of RPRE on *E. coli*

After *E. coli* O101 was cultured with concentrations of RPRE from 0–128 mg/mL, no significant differences in bacterial growth rate were observed, but the growth of bacteria treated with RPRE was approximately 15% lower than that of the control. However, even at the highest concentration of RPRE (128 mg/mL), the bacterial growth rate still exceeded 70% (Table 2). This finding indicates that RPRE has a weak inhibitory effect on the proliferation of *E. coli*.

**Table 2.** Effect of *Radix Paeoniae Rubra* aqueous extract (RPRE) on the proliferation of *E. coli* O101

RPRE (mg/mL)	Bacterial growth rate (%)
0	100.00 ± 0.00
1	86.75 ± 0.37
2	87.68 ± 0.33
4	84.66 ± 0.15
8	85.36 ± 0.40
16	85.15 ± 0.19
32	85.81 ± 0.40
64	75.71 ± 0.33
128	70.61 ± 0.36

### Sensitivity of *E. coli* to commonly used antibiotics

The microdilution method was used to detect the sensitivity of the bacteria to the commonly used antibiotics. Bacterial growth was considered inhibited if the growth rate was less than 10% of the control group (without antimicrobials). The results showed that the MIC of gentamicin, tetracycline, doxycycline, florfenicol and enrofloxacin for *E. coli* O101 was 16 μg/mL; the MICs of streptomycin and cephalexin were 32 μg/mL and 64 μg/mL, respectively; the MIC of kanamycin and spectinomycin was 128 μg/mL; and the MIC of tilmicosin and sulphadimethoxine was greater than 256 μg/mL. According to the standards for antimicrobial susceptibility and the relationship between the MIC determined and the specified resistance concentration published by the CLSI (5), *E. coli* O101 was classified as having low resistance to gentamicin, spectinomycin, tetracycline, doxycycline, sulphadimethoxine and florfenicol; intermediate resistance to kanamycin, streptomycin and cephalexin; and high resistance to tilmicosin and enrofloxacin (Table 3). This indicates that the *E. coli* O101 used in this study is a multi-drug resistant strain.

**Table 3.** Minimum inhibitory concentrations (MIC) of antibiotics against *E. coli* O101 used with *Radix Paeoniae Rubra* aqueous extract

Type	Antibiotic	MIC (μg/mL)	CLSI M100 33^rd^ edition *E. coli* breakpoint (μg/mL)			Low resistance (R ≤ MIC < 2R) (μg/mL)†	Moderate resistance (2R ≤ MIC < 4R) (μg/mL)†	High resistance (MIC ≥ 4R) (μg/mL)†	Noted resistance
			S	I	R				
Aminoglycosides	Gentamicin	16	≤4	8	≥16	16–31	32–63	≥64	Low
	Kanamycin	128	≤16	32	≥64	64–127	128–255	≥256	Moderate
	Streptomycin	32	—	—	—				Moderate‡
	Spectinomycin	128	≤32	64	≥128	128–255	256–511	≥512	Low
Tetracyclines	Tetracycline hydrochloride	16	≤4	8	≥16	16–31	32–63	≥64	Low
	Doxycycline	16	≤4	8	≥16	16–31	32–63	≥64	Low
Cephalosporins	Cephalexin	64	≤8	16	≥32	32–63	64–127	≥128	Moderate
Sulphonamides	Sulphadimethoxine§	>256	≤256	—	≥512	512–1,023	1,024–2,047	≥2,048	Low
Macrolides	Tilmicosin	>256	—	—	≥16	16–31	32–63	≥64	High
Amphenicols	Florfenicol	16	≤8	—	≥16	16–31	32–63	≥64	Low
Quinolones	Enrofloxacin	16	≤1	—	≥4	4–7	8–16	≥16	High

1 S – susceptible; I – intermediate; R – resistant;

† – based on CLSI breakpoints;

‡ – based on the CLSI breakpoint for gentamicin as a same-class antimicrobial in the absence of one for streptomycin against *E. coli*;

§ – an MIC of >256 μg/mL was noted (the highest concentration tested); bacterial growth was only inhibited by ~22% at 256 μg/mL. Therefore, *E. coli* O101 was classified as low-level resistant. For drugs without a CLSI specified resistance concentration below which *E. coli* resistance is known, but with a determined MIC higher than the breakpoints of similar drugs, *E. coli* O101 was marked as resistant

### Effect of RPRE on the inhibition of *E. coli* proliferation by antibiotics

**Fig. 1.**
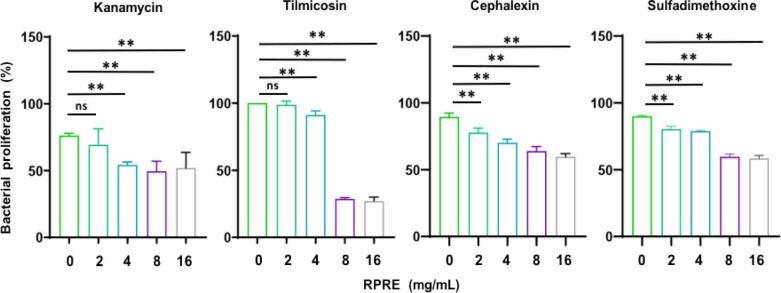
Effect of *Radix Paeoniae Rubra* aqueous extract (RPRE) on the antimicrobial resistance of *E. coli* O101. Growth of untreated control *E. coli* was taken as 100%, and bacterial growth in the treatment groups was calculated in relation to that benchmark. Data are expressed as mean ± SD (n = 3). ** – P < 0.01; ns – not significant (P > 0.05)

Based on the chemical properties of antibiotics, one aminoglycoside, one cephalosporin, one sulphonamide and one macrolide were selected for testing (kanamycin, cephalexin, sulphadimethoxine and tilmicosin, respectively). *Escherichia coli* showed low resistance to sulphadimethoxine, moderate resistance to cephalexin and kanamycin and high resistance to tilmicosin. The results showed that the combination of RPRE with these antibiotics inhibited the proliferation of *E. coli* in a concentration-dependent manner (RPRE at concentrations of 4 mg/mL and above enhanced the antibacterial effect of all four antibiotics) (Fig. 1). This suggests that RPRE has the ability to enhance the susceptibility of resistant *E. coli* to antibiotics, even to those it resists best, *e.g*. tilmicosin, especially at concentrations equal to or greater than 8 mg/mL (Fig. 1).

### Effect of RPRE on the secretion of *E. coli* virulence factor proteins

**Fig. 2.**
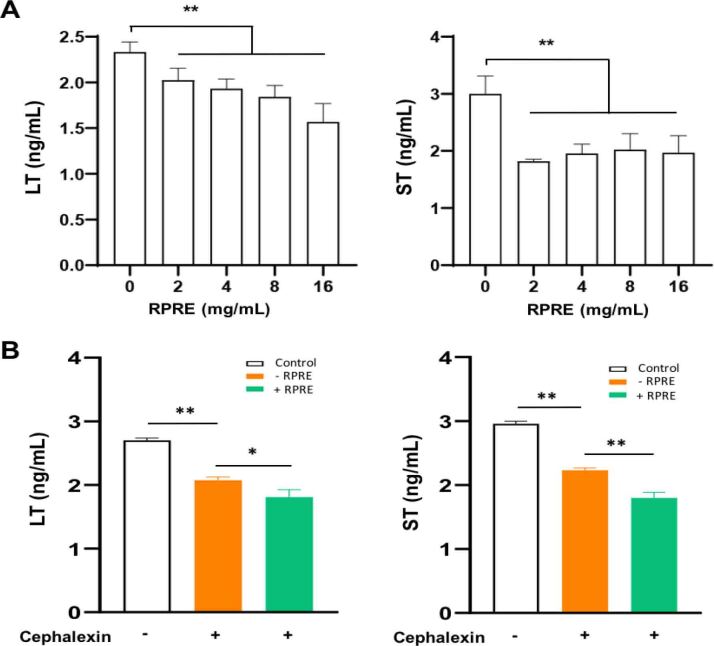
Effect on the secretion of *E. coli* O101 virulence factor proteins of 24-h incubation in *Radix Paeoniae Rubra* aqueous extract (RPRE). A – secretion with RPRE at concentrations ranging 0–16 mg/mL and without cephalexin; B – secretion with RPRE at 4 mg/mL and cephalexin at 4 μg/mL. Data are expressed as mean ± SD (n = 3). * – P < 0.05; ** – P < 0.01

After the addition of different concentrations of RPRE to the *E. coli* culture medium, the secretion of the virulence factors heat-labile enterotoxin (LT) and heat-stable enterotoxin (ST) by *E. coli* decreased in a concentration-dependent manner (Fig. 2A), suggesting that RPRE could reduce the bacterium’s pathogenicity by this inhibition. Cephalexin also had a certain inhibitory effect on LT and ST secretion by *E. coli*: when the bacterium was cultured in the medium containing RPRE at 4 mg/mL in the presence of cephalexin, this was further inhibited (P < 0.01) (Fig. 2B). The results demonstrated that RPRE acted synergistically with an antimicrobial drug to inhibit *E. coli* O101 virulence factor protein secretion.

### Effect of RPRE in combination with antibiotics on the formation of *E. coli* biofilm

**Fig. 3.**
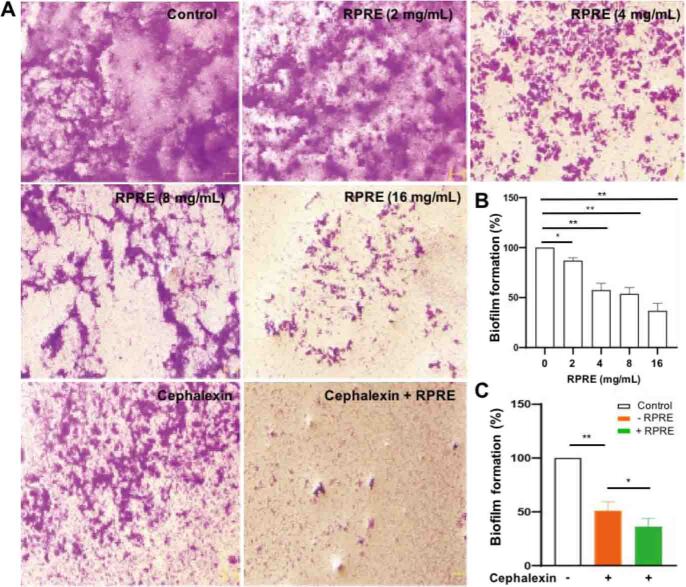
Effect on the biofilm formation of *E. coli* O101 of three-day incubation in *Radix Paeoniae Rubra* aqueous extract (RPRE) with or without treatment with cephalexin at 32 μg/mL. A – micrographs of crystal violet–stained biofilm; B and C – biofilm formation by untreated *E. coli* was taken as 100%, and the biofilm formation in the treatment groups was calculated in relation to that benchmark. Data are expressed as mean ± SD (n = 3). * – P < 0.05; ** – P < 0.01

The biofilm of *E. coli* O101 is closely related to its drug resistance and pathogenicity. The control group results showed the bacterial biofilm existing in sheets with the films tightly connected to each other. After treatment with low concentrations of RPRE (2–4 mg/mL), fissures between the biofilms can be observed. As the concentration of RPRE increased, the biofilms became gradually sparser, and after treatment with high concentrations of RPRE (8–16 mg/mL), they were mostly scattered in small plaque form (Fig. 3A). Quantitative analysis shows that as the concentration of RPRE increased, the OD values of the RPRE treatment group significantly decreased (P < 0.01) (Fig. 3B), suggesting that RPRE inhibited formation of the bacterial biofilm. Cephalexin also had a certain inhibitory effect on the formation of the biofilm, and when RPRE at 4 mg/mL was combined with cephalexin in the treatment, the formation of the bacterial biofilm was further inhibited (P < 0.01) (Fig. 3A and 3C). The results fully demonstrated that RPRE can act synergistically with an antimicrobial drug to inhibit the formation of the biofilm of the bacteria.

### Effect of RPRE on expression of *E. coli* efflux pump genes

**Fig. 4.**
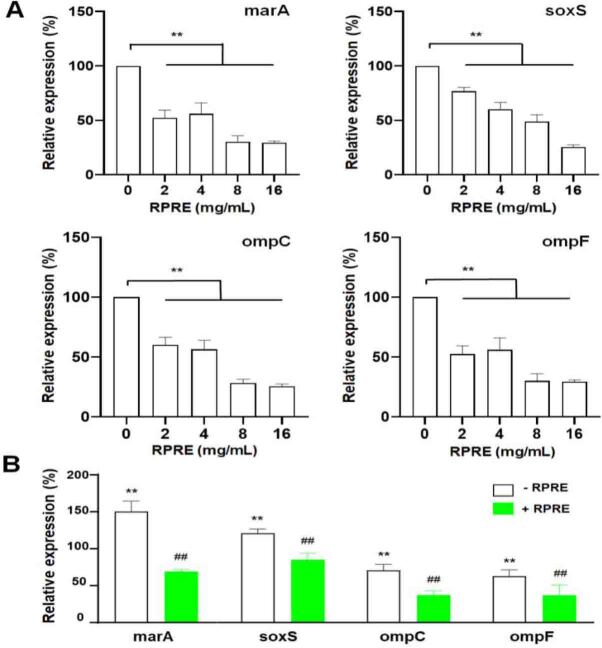
Effect on the expression of *E. coli* O101 efflux pump and outer membrane porin genes of 24-h incubation in *Radix Paeoniae Rubra* aqueous extract (RPRE) with or without treatment with cephalexin at 32 μg/mL. A – expression with RPRE in concentrations ranging 0–16 mg/mL; B – expression with RPRE at 4 mg/mL and cephalexin at 32 μg/mL. Expression by untreated *E. coli* was taken as 100%, and the expression in the treatment groups was calculated in relation to that benchmark. Data are expressed as mean ± SD (n = 3). ** – significant difference compared with the blank control (P < 0.01); ## – significant difference compared with the group without RPRE treatment (P < 0.01)

With rising RPRE concentrations, the relative expression levels of the *marA* and *soxS* genes fell significantly (P < 0.01). After culture with RPRE at 0–16 mg/mL, *E. coli* expressed *marA* 36.85–77.00% and *soxS* 20–70% less (Fig. 4A). When cephalexin was used alone, the relative expression of these genes was higher, which indicated that the antibiotic induced their expression. However, when *E. coli* was cultured in the medium containing 4 mg/mL of RPRE in addition to cephalexin, the increase in transcription levels of *marA* and *soxS* induced by the antimicrobial was changed to a decrease to below the untreated control bacteria transcription level (Fig. 4B), indicating that RPRE inhibited the transcription of efflux pump genes. Thereby, RPRE may inhibit the bacterial efflux function.

### Effect of RPRE on expression of outer membrane porin genes

This study also examined the effect of RPRE on the transcription levels of the *ompC* and *ompF* genes that encode bacterial outer membrane porins. As the RPRE concentration was stepped up, the expression of the genes decreased in a dose-dependent manner (P < 0.01) (Fig. 4A). The relative expression level of *ompC* decreased by 17.78–74.49% and that of *ompF* by 35.65–70.59%. Notably, treatment with cephalexin alone could also reduce these genes’ transcription levels (P < 0.01), and when combined with 4 mg/mL of RPRE, the expression of *ompC* and *ompF* further decreased by 19.16% and 18.59%, respectively (P < 0.01) (Fig. 4B), indicating that RPRE inhibited the transcription levels of the outer membrane porin genes *ompC* and *ompF* and had a synergistic effect with the antibiotic.

## Discussion

Traditional Chinese medicinal herbs have been recognised with potential in reducing bacterial pathogenicity and resistance to antibiotics (10, 12, 27). This study reveals the role of RPRE, which is from a widely used TCM herb, in restoring the susceptibility of MDR bacteria to antibiotics. Although the bacteriostatic effect of RPRE alone on drug-resistant *E. coli* is weak, and its direct antibacterial activity is significantly lower than that of other reported TCM herbs, such as *Polygonum multiflorum* extract (with an MIC of 0.2 mg/mL–1.00 mg/mL for Gram-positive strains and 0.75 mg/mL–19.50 mg/mL for Gram-negative strains) and *Sophora flavescens* ethanol extract (with an MIC of 8 μg/mL for methicillin-resistant *Staphylococcus aureus*) (18, 24), it can significantly enhance the germicidal effect of antimicrobial drugs on MDR bacteria when combined with kanamycin and cephalexin. This phenomenon indicates that the mechanism of action of RPRE may differ from the multi-target mode of direct antibacterial action, and that its core function is more likely to be regulating the pathways related to bacterial resistance rather than directly inhibiting bacterial proliferation.

Recent studies have shown that the berberine, flavonoids, alkaloids, phenols, quinones, and polydatin components of *Scutellaria baicalensis* Georgi, *Reseda odorata* L. and *Camellia sinensis* (L.) Kuntze or TCM preparations like YinHuaPingGanKeli granules can reverse bacterial antimicrobial resistance by inhibiting bacterial efflux pump activity, disrupting biofilm formation or regulating the expression of drug resistance genes (7, 8, 31, 32). For example, resveratrol, a compound of plant origin, inhibited the biofilm formation of avian pathogenic *E. coli* (17), and eugenol combined with colistin enhanced the antibacterial activity of antibiotics against colistin-resistant *E. coli*, while eugenol combined with either cefotaxime or ciprofloxacin was active against quinolone-resistant Enterobacteriaceae bacteria (25). It is known that the formation of bacterial biofilms and the expression levels of outer membrane porins and efflux pump regulators are related to not only the pathogenicity of bacteria but also their drug resistance (28, 29). *Radix Paeoniae Rubra* aqueous extract, when used in combination with antibiotics, did not show selective synergy with specific types of antibiotics and had the effect of reducing resistance to macrolides, aminoglycosides, cephalosporins and sulphonamides, suggesting that RPRE may affect bacterial sensitivity to antibiotics through a broad-spectrum mechanism (including the inhibition of outer membrane porin expression or interference with drug resistance regulatory systems) (13). This study confirms that RPRE can inhibit the transcription of the outer membrane porin–encoding genes *ompC* and *ompF*, as well as the efflux pump genes *marA* and *soxS*, which is important because the production levels of the proteins encoded by these genes are closely related to the membrane permeability and antibiotic uptake efficiency of the bacteria (2, 33). We speculated that RPRE may increase the intracellular accumulation of antibiotics by inhibiting the expression of the genes related to the outer membrane barrier function, resulting in a greater microbicidal effect on MDR bacteria and thereby raising the low susceptibility of drug-resistant bacteria to antimicrobial drugs. This resistance-lowering effect could extend the lifespans of the available antibiotics and aid the development of new drugs (21).

It has been found that TCM preparations and their main components can reduce the pathogenicity of bacteria by interfering with the production of virulence factors. In Enterobacteriaceae, ST and LT are the main virulence factors, and they primarily act on the host’s intestinal epithelial cells (28), causing diarrhoea and dehydration (1, 3). The current study found that RPRE could also inhibit the production of ST and LT in Gram-negative *E. coli*, especially when combined with cephalexin. This inhibitory effect of RPRE could mitigate the damage of virulence factors to tissues and organs to reduce the pathogenicity of bacteria (16).

The more-pursued strategy to combat MDR bacteria relies heavily on the research and development of new antibiotics, which not only requires a long period but also can hardly avoid the occurrence of new resistance (14, 20, 26). The combination of RPRE with antibiotics offers a more sustainable and feasible alternative solution; the propagation of resistant mutants could be reduced by applying sub-lethal stress to regulate bacterial resistance. *Radix Paeoniae Rubra* aqueous extract could function as an antibiotic adjuvant that modulates bacterial resistance, decelerating resistance evolution trajectories and extending the clinical lifespan of the currently available antimicrobial drugs. It is expected to be used clinically to treat MDR bacterial infections, permitting reduction of the dosage of antibiotics.

## Conclusion

*Radix Paeoniae Rubra* aqueous extract can increase the susceptibility of MDR bacteria to antibiotics by inhibiting the expression of bacterial efflux pump genes, altering the permeability of the outer membrane pores and affecting the formation of biofilms, and can suppress bacterial virulence factors to decrease the pathogenicity of the bacteria.
